# New insights into the genome repetitive fraction of the Antarctic bivalve *Adamussium colbecki*

**DOI:** 10.1371/journal.pone.0194502

**Published:** 2018-03-28

**Authors:** Maria Assunta Biscotti, Marco Barucca, Adriana Canapa

**Affiliations:** Dipartimento di Scienze della Vita e dell’Ambiente, Università Politecnica delle Marche, Ancona, Italy; Birla Institute of Technology and Science, INDIA

## Abstract

Repetitive DNA represents the major component of the genome in both plant and animal species. It includes transposable elements (TEs), which are dispersed throughout the genome, and satellite DNAs (satDNAs), which are tandemly organized in long arrays. The study of the structure and organization of repetitive DNA contributes to our understanding of genome architecture and the mechanisms leading to its evolution. Molluscs represent one of the largest groups of invertebrates and include organisms with a wide variety of morphologies and lifestyles. To increase our knowledge of bivalves at the genome level, we analysed the Antarctic scallop *Adamussium colbecki*. The screening of the genomic library evidenced the presence of two novel satDNA elements and the CvA transposon. The interspecific investigation performed in this study demonstrated that one of the two satDNAs isolated in *A*. *colbecki* is widespread in polar molluscan species, indicating a possible link between repetitive DNA and abiotic factors. Moreover, the transcriptional activity of CvA and its presence in long-diverged bivalves suggests a possible role for this ancient element in shaping the genome architecture of this clade.

## Introduction

Repetitive DNA has been identified in all major taxonomic groups of both plant and animal species, in many cases representing the majority of the DNA content in the genome [1–3]. Repetitive elements include transposable elements (TEs), which are dispersed throughout the genome, and satellite DNAs (satDNAs), which are tandemly organized in long arrays.

TEs are characterized by their ability to proliferate and move throughout the genome. They are classified as retrotransposons or DNA transposons depending on whether there is an RNA intermediate step in the transposition mechanism. TEs can also be divided into autonomous or non-autonomous based on their ability to use their own enzymes or those synthetized by other transposable elements to carry out transposition. TE abundance is correlated with genome size, explaining the C-value paradox, that is, the lack of correlation between DNA content and organism complexity in different species [3]. TEs can play a role in the reorganization of the genome through chromosomal rearrangements such as duplications, inversions, and translocations as well as through molecular domestication, a phenomenon that gives rise to new coding genes and regulatory elements [4–7]. Moreover, TEs show lineage-specific diversity in terms of composition, content and age, contributing to genome plasticity and the evolution of the host genome [8].

SatDNAs are mainly located in telomeric, centromeric or pericentromeric regions. The role of this fraction of repetitive DNA is not completely understood, although several structural and functional roles have been proposed [9]. For example, localization at the centromere suggests potential involvement in centromeric DNA packaging [10,11], chromosome segregation during mitosis and meiosis, pairing of homologous chromosomes, sister chromatid attachment, and kinetochore formation [12]. Moreover, satDNA transcripts play a role in heterochromatin formation and maintenance at both the centromere and telomere, with a consequent impact on karyotype evolution [9]. The comparison of the nature and localization of satDNAs between species reveals that some repetitive DNAs are extremely well conserved [13–15], while others are highly variable even between closely related species [16–17]. Moreover, satDNAs may share sequence similarity to TEs, indicating that complex mutual relationships can determine their evolution, influencing the genome architecture [18–19].

Overall, different modes of repetitive DNA evolution in various organisms underlie the key role of repetitive DNA in shaping genomes. In ectothermal organisms, environmental variables can drive adaptive evolution and consequently influence the evolution of repetitive DNA. Indeed, recent papers have demonstrated a correlation between repetitive DNA and environmental temperature [20,21].

Molluscs represent one of the largest groups of invertebrates and include organisms with a wide variety of morphologies and lifestyles. However, to date, only four molluscan species have been sequenced [22–25], and the presence and characteristics of satellite elements and transposons have been analysed in a limited number of species [14,15,18,22,24,26–48]. Indeed, satDNAs, given their tandem organization and sequence homogeneity, represent the major unassembled component in the sequenced genomes owing to difficulties in the assembly of repetitive DNA [49].

To increase our knowledge of bivalves at the genome level, we analysed the scallop *Adamussium colbecki*, a marine bivalve belonging to the Pectinidae family that is adapted to cold Antarctic waters.

The screening of the genomic library evidenced the presence of two novel satDNA elements and the CvA transposon. The interspecific investigation performed in this study demonstrated that one of the two satDNAs isolated in *A*. *colbecki* is widespread in Antarctic species, indicating a link between repetitive DNA and abiotic factors. Finally, the transcriptional activity of CvA and its presence in divergent bivalves suggests a possible role of this element in shaping the genome architecture of these molluscan species.

## Material and methods

### Identification of repetitive elements in *A*. *colbecki*: Library construction and screening

The specimens of *A*. *colbecki* were collected in Terra Nova Bay, Ross Sea, in the austral summer of 2013–2014 during an Italian expedition to the Antarctic (permit PdR 2009/A1.10, issued under “Programma Nazionale di Ricerche in Antartide” (PNRA)). Genomic DNA was extracted from muscle using DNAzol reagent (Invitrogen, Carlsbad, CA) following the manufacturer’s instructions. Following the strategy described by Biscotti et al. [40], 20 μg of *A*. *colbecki* DNA were digested overnight at 37°C with the restriction enzyme *Eco*RI (Fermentas, Vilnius, LI) according to the manufacturer’s instructions. After electrophoresis on a 1.5% agarose gel, the region containing fragments with a molecular weight between 100 bp and 1,000 bp was excised and purified with a QIAquick Gel Extraction Kit (QIAGEN, Hilden, DE) and cloned into the pZErO-2.1 vector with a Zero background/Kan Cloning Kit (Invitrogen, Carlsbad, CA).

Colonies were transferred onto a positively charged membrane (Bio-Rad, Hercules, CA) and lysed by washing with 25 ml of denaturing solution (1.5 M NaCl, 0.5 M NaOH) for 5 min, followed by two washes with 25 ml of neutralizing solution (0.5 M Tris-HCl, 1.5 M NaCl) for 5 min each and a further 5 min in 25 ml of 2x SSC. DNA was then fixed by baking at 120°C for 30 min.

To identify colonies with inserts corresponding to repetitive sequences in the scallop genomes, 3 μg of DNA of *A*. *colbecki* were thermally cleaved (4 min, 120°C), and fragments were marked with digoxigenin-dUTP using the random primer labelling method. Hybridization was performed overnight at 42°C, and the detection of hybridization signals was obtained following the DIG High Prime DNA labelling and Detection Starter Kit (Roche, Indianapolis, IN) manufacturer’s instructions. Colonies showing stronger signals were selected and sequenced. The sequence analysis identified two novel satDNAs named Ac4p1 (*A**damussium* *c**olbecki* 4p1) and Ac4p3 (*A**damussium* *c**olbecki* 4p3) and four sequences with similarity to the CvA transposon elements of other scallops, such as *Chlamys farreri* (accession number JN703459, EF207401, DT717291, DT719341, DT716679, JM413173, JM423271, EI407096), *Pecten maximus* (accession number AM279154), *Mizuhopecten yessoensis* (GT565599, GT569360, GT565389), and *Patinopecten magellanicus* (GAD601008570, GAD601008569, GAD601002094, GAD601002092). To demonstrate that the sequences isolated were CvA, we amplified the region upstream of the repeated core. The forward primer 5’-AGAGGGCCTGTATCGCTCACC-3’ was designed based on the alignment of the non-repetitive region of *C*. *farreri* CvA sequences available in GenBank, and the reverse primer 5’-GGTCTGGAGCATTGTTAGGTGAAG-3’ was designed based on the sequences obtained here corresponding to the repetitive region of the element. The amplification reaction was performed on total RNA isolated from gonads and muscle of *A*. *colbecki* (as described below) under the following conditions: 2 min at 94°C; 30 cycles at 94°C for 1 min, 48°C for 1 min, and 72°C for 1 min; and 10 min at 72°C. The amplified product was cloned and sequenced. The repetitive sequences isolated were deposited in GenBank (accession Nos: MG882404- MG882410).

### Search for Ac4p3 in *A*. *colbecki*, *Chlamys islandica* and *C*. *varia*

To test the conservation of the Ac4p3 sequence in *C*. *islandica* and *C*. *varia*, a degenerate primer pair (forward: 5’-CTATAWSTCATCMACTACAGGTC-3’; reverse: 5’-TAGGARTYVTACTWTGTGGTAG-3’) was designed based on the consensus sequence obtained from the repeats present in the inserted sequence. The conditions for the PCR reactions were as follows: 2 min at 94°C; 20 cycles at 94°C for 1 min, 52°C for 1 min, and 72°C for 1 min; and 10 min at 72°C. Amplicons were purified, cloned, and sequenced. The presence of Ac4p3 in *C*. *varia* was also tested by Southern blotting (as described below) using the Ac4p3 from *A*. *colbecki* as a probe.

### Transcriptional activity of Ac4p3 and the CvA element

Total RNA was isolated from *A*. *colbecki* and *C*. *islandica* gonad and muscle (tissues collected from species of interest during the sampling) with TRIzol reagent (Invitrogen, Carlsbad, CA) and treated with amplification grade DNase according to the manufacturer’s instructions (Sigma-Aldrich, Darmstadt, DE). The cDNA was obtained using SuperScript III First-Strand Synthesis SuperMix (Invitrogen, Carlsbad, CA). The transcriptional activity of Ac4p3 in *A*. *colbecki* and *C*. *islandica* was detected using the above degenerate primer pair under the following conditions: 2 min at 94°C; 30 cycles at 94°C for 1 min, 48°C for 1 min, and 72°C for 1 min; and 10 min at 72°C. The transcriptional activity of the CvA element in *A*. *colbecki* was tested using the above primer pair under the same conditions.

### Southern blotting and dot blot analyses

Genomic DNA digested with *Eco*RI was electrophoresed on a 1.5% agarose gel and transferred onto a positively charged membrane for Southern blotting analyses (Bio-Rad, Hercules, Calif., USA). The clones of interest were amplified and labelled with digoxigenin-dUTP and used as probes for hybridization, which was performed overnight at 47°C for clones Ac4p1 and Ac19p1 and at 46°C for clone Ac4p3. Stringency washes and detection of signals were performed following the DIG High Prime DNA labelling and Detection Starter Kit (Roche, Indianapolis, IN) manufacturer’s instructions. The probes used for Southern blotting were also used in dot blot analyses. For qualitative analyses, 100 ng of each genomic DNA was spotted and fixed (120°C, 30 min) onto a positively charged membrane. For quantitative dot blot analyses, 1 ng, 0.5 ng, 0.25 ng, 0.12 ng, 0.06 ng, and 0.03 ng of genomic DNA were used. Hybridization and detection were carried out as specified for Southern blotting.

### Bioinformatic analyses

The obtained sequences were compared with the EMBL and GenBank databases using the NCBI BLAST server [50] and with the molluscan whole genomes available in Ensembl Metazoa (http://metazoa.ensembl.org/Multi/blastview). Features related to transposable elements were searched in Repbase using Censor (http://www.girinst.org/censor/) [51]. The detection of internal repeats was performed through Dotlet (http://myhits.isb-sib.ch/cgi-bin/dotlet) [52], while tRNAscan-SE Search Server (http://lowelab.ucsc.edu/tRNAscan-SE/) [53,54] was used to search for motifs corresponding to the internal promoters of tRNAs (A and B boxes).

## Results

Genomic DNA from *A*. *colbecki* was digested with the restriction enzyme *Eco*RI and cloned, and the resultant colonies were hybridized with labelled genomic DNA from the Antarctic scallop. The screening of the *A*. *colbecki* genomic library yielded 20 colonies showing a strong hybridization signal. The sequence analysis identified two novel satDNAs and a sequence with homology to the CvA transposon element. Clones with short inserts and no subrepeat structure were not further analysed.

**Fig 1:**
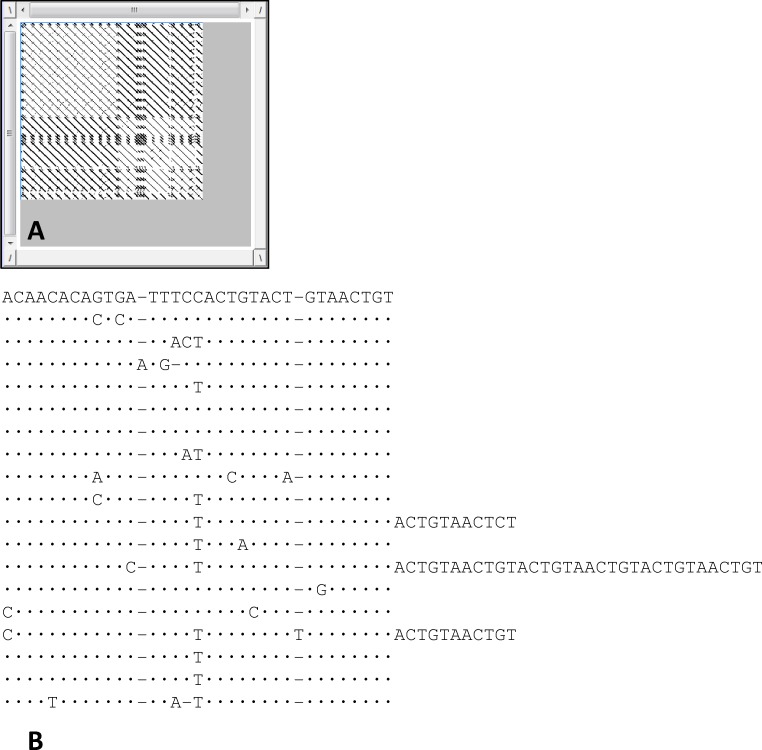
Dotlet plot and alignment of repeats present in the Ac4p1 inserted sequence. A: Dotlet plot showing the repeats present in the Ac4p1 sequence; B: alignment of 19 complete repeat elements identified in the Ac4p1 inserted sequence.

Two insert sequences, of 726 bp (61.98% of A/T content) and 237 bp (62.02% of A/T content), were sequenced. Dotlet analysis (Fig 1A) of the first sequence, named Ac4p1, showed 19 complete repeat elements, 16 of which are characterized by an average length of approximately 33 bp. This 33 bp repeat seems to originate from a subrepeat of approximately 11 bp that has undergone greater diversification (Fig 1B). The other three repeats have a longer length due to the presence of a variable number of subrepeats. The distance matrix built on the alignment of the repeats isolated highlighted a similarity rate of 90.09%, indicating a high level of homogenization.

**Fig 2:**
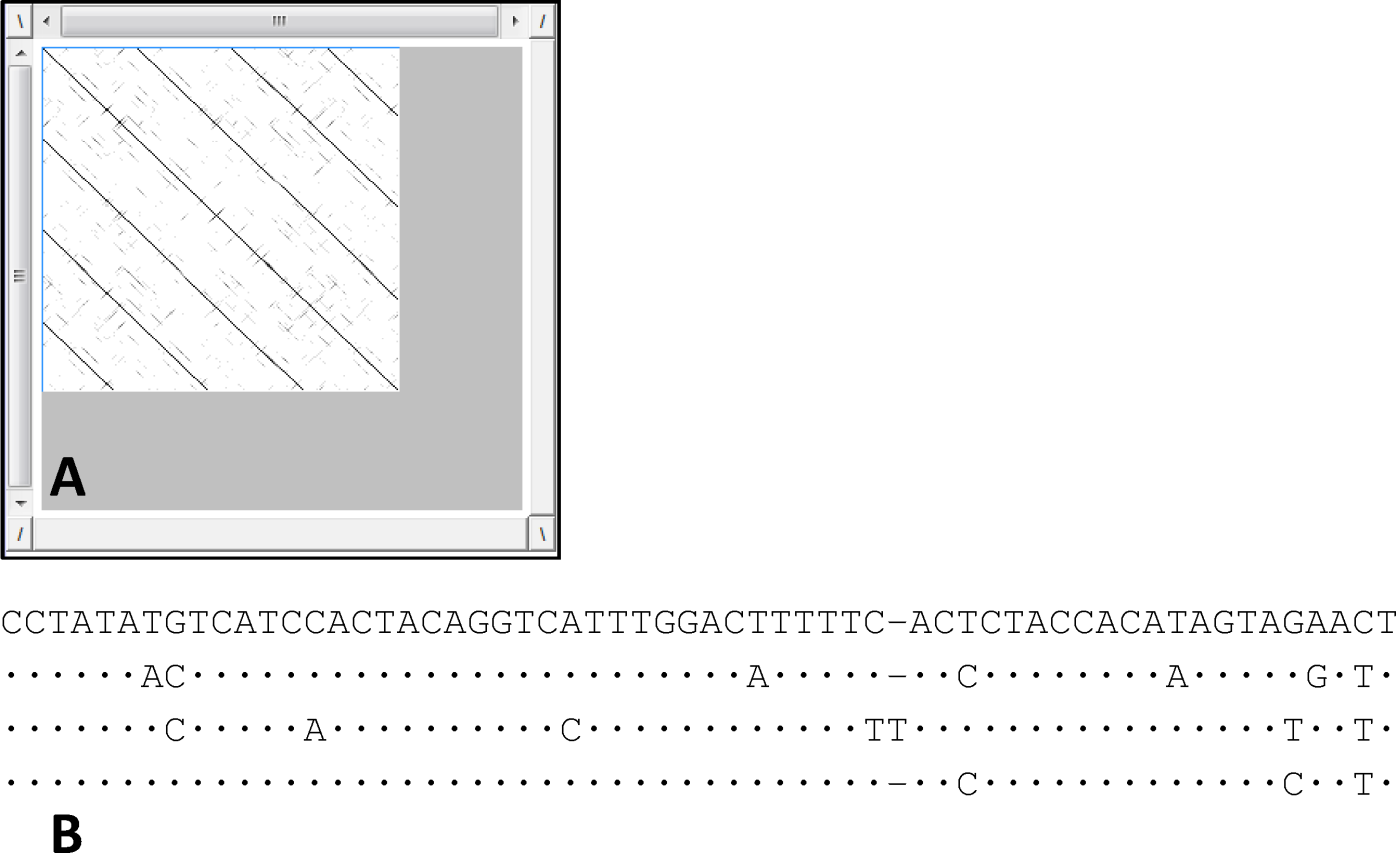
Dotlet plot and alignment of repeats present in the Ac4p3 inserted sequence. A: Dotlet plot showing the repeats present in the Ac4p3 sequence; B: alignment of four repeat elements identified in the Ac4p3 inserted sequence.

Dotlet analysis of the other inserted sequence, named Ac4p3, showed four repeat elements of approximately 60 bp in length each (Fig 2A). The distance matrix built on the alignment of the isolated repeats (Fig 2B) highlighted an average percent similarity of 81.87%. For both sequences, comparison with GenBank and Repbase databases did not reveal any significant similarity to known sequences.

**Fig 3:**
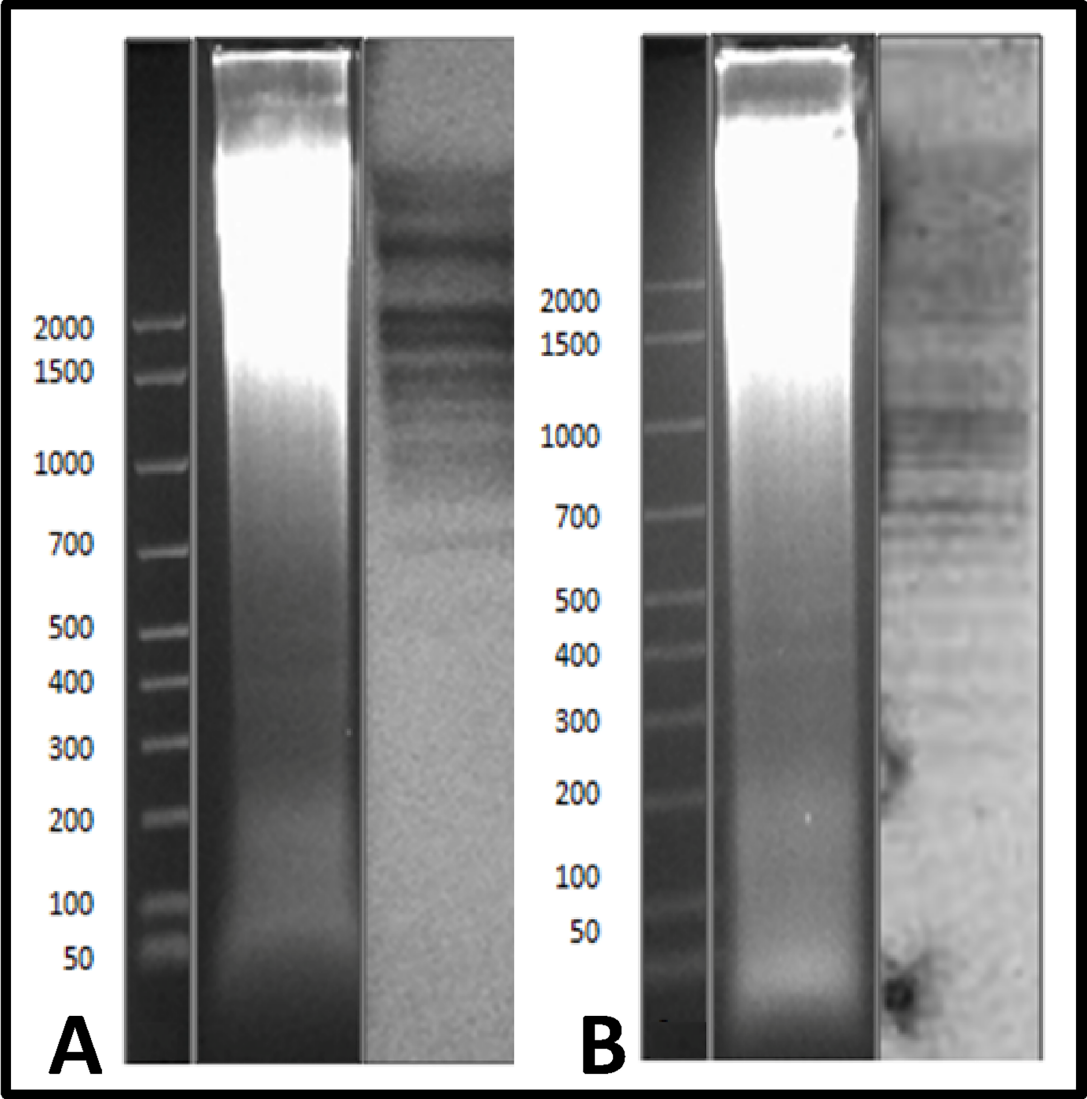
Southern blotting analyses using Ac4p1 and Ac4p3 probes. A: Genomic DNA of *A*. *colbecki* digested with *Eco* RI and Southern blot hybridization with the Ac4p1 probe; B: Genomic DNA of *A*. *colbecki* digested with *Eco* RI and Southern blot hybridization with the Ac4p3 probe.

The genomic organization of the two clones of interest was investigated by Southern blotting analyses. For Ac4p1, the hybridization band pattern suggested a complex organization (Fig 3A) due to the presence of a variable number of subrepeats, as also evidenced by dotlet analysis. For Ac4p3, the hybridization pattern showed a characteristic ladder of tandem elements (Fig 3B) with the lowest molecular weight band at approximately 250 bp, probably representing the monomer.

**Fig 4:**
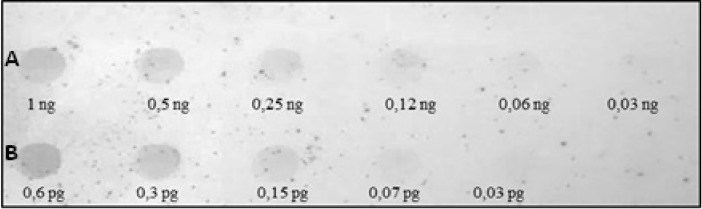
Quantitative dot blot hybridization using the Ac4p1 probe. In row A, increasing amounts of *A*. *colbecki* genomic DNA are spotted from left to right. In row B, increasing amounts of the Ac4p1 sequence are spotted from left to right.

**Fig 5:**
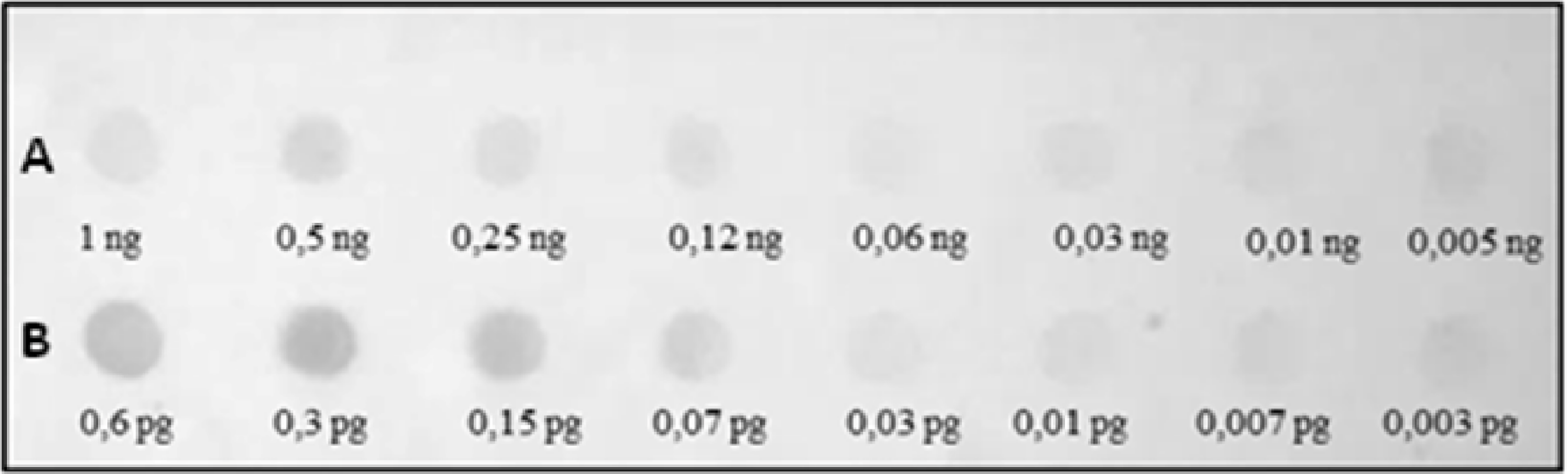
Quantitative dot blot hybridization using the Ac4p3 probe. In row A, increasing amounts of *A*. *colbecki* genomic DNA are spotted from left to right. In row B, increasing amounts of the Ac4p3 sequence are spotted from left to right.

Different abundances of the identified repeats were evidenced by quantitative dot blot analyses. Specifically, the sequence Ac4p1 represents less than 0.06% of the *A*. *colbecki* genome (Fig 4), while the sequence Ac4p3 is approximately 0.01% (Fig 5).

**Fig 6:**
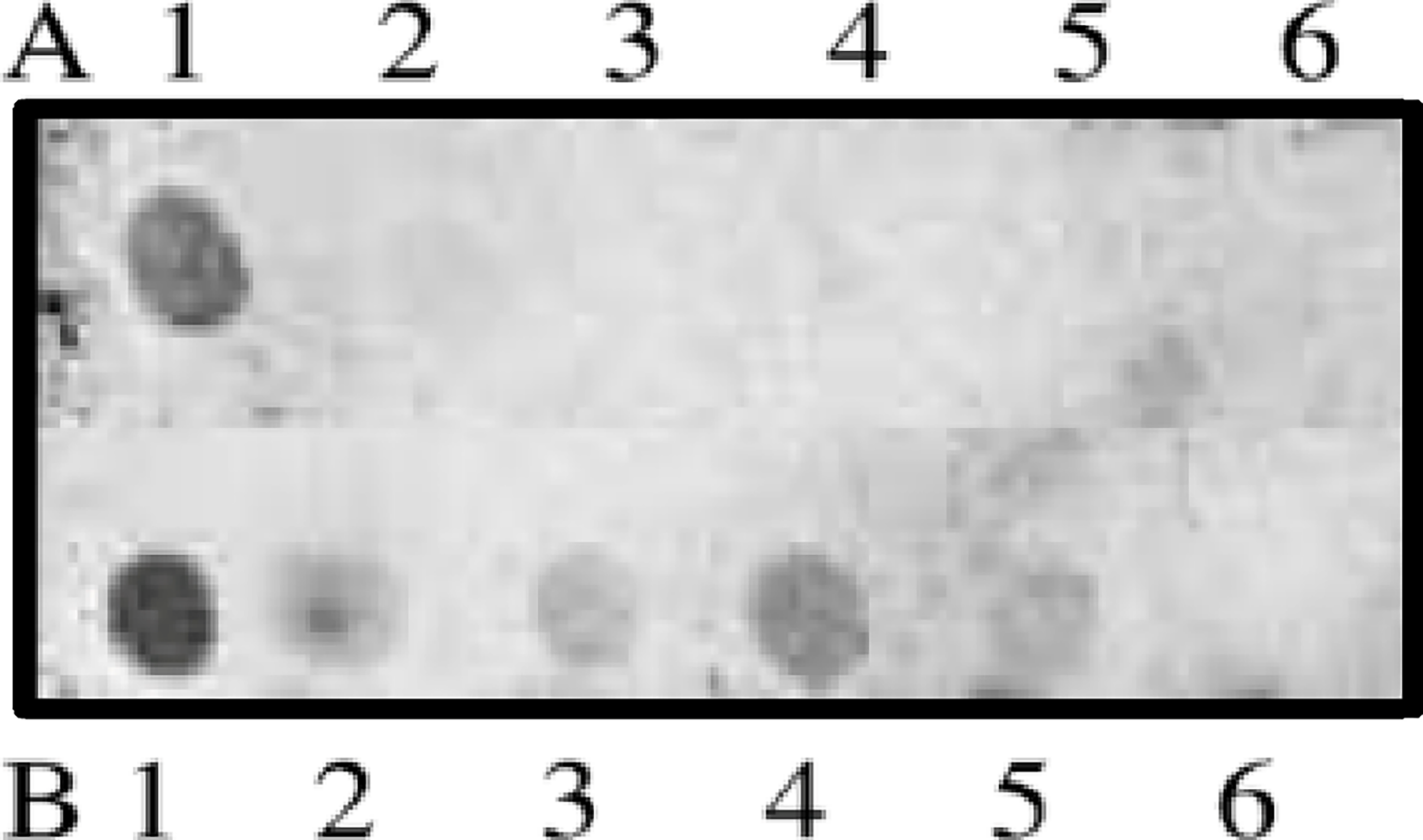
Qualitative dot blot analysis using the Ac4p3 probe. In row A, the genomes of pectinid species are spotted: 1. *A*. *colbecki*, 2. *C*. *islandica*, 3. *M*. *nobilis*, 4. *G*. *pallium*, 5. *P*. *maximus*, 6. *A*. *opercularis*. The first two species are adapted to polar environments, and the others are adapted to temperate regions. In row B, marine Antarctic species other than the species of interest are analysed: 1. *A*. *colbecki*, 2. *Buccinum sp*., 3. *N*. *eatoni*, 4. *L*. *elliptica*, 5. *T*. *bernacchii*, 6. negative control.

The presence of the identified sequences was also investigated in the genomes of five other pectinids, *Aequipecten opercularis*, C*hlamys islandica*, *Gloripallium pallium*, *Pecten maximus*, and *Mimachlamys nobilis*, and in the polar species *Buccinum sp*. (Gastropoda), *Neobuccinum eatoni* (Gastropoda), *Laternula elliptica* (Bivalvia) and *Trematomus bernacchii* (Teleostei). The qualitative dot blot analyses showed the presence of element 4p3 in all species from the polar regions, while no signal was detected in the species of scallops from temperate regions (Fig 6).

To verify whether the presence of the Ac4p3 sequence in polar organisms may be related to the cold environment, the Arctic species *C*. *islandica* and the temperate species *C*. *varia*, two scallops more phylogenetically related to each other than to *A*. *colbecki* [55,56], were investigated. PCR analysis generated a multiband product in *C*. *islandica*, and the lowest molecular weight band (~200 bp) was purified and sequenced. The obtained sequence showed a similarity of 84.51% to that isolated in *A*. *colbecki*, confirming the presence of this element in the Artic scallop. The PCR did not produce any amplification product from *C*. *varia*. Moreover, Southern hybridization carried out on digested DNA from this bivalve using Ac4p3 as a probe was negative.

**Fig 7:**
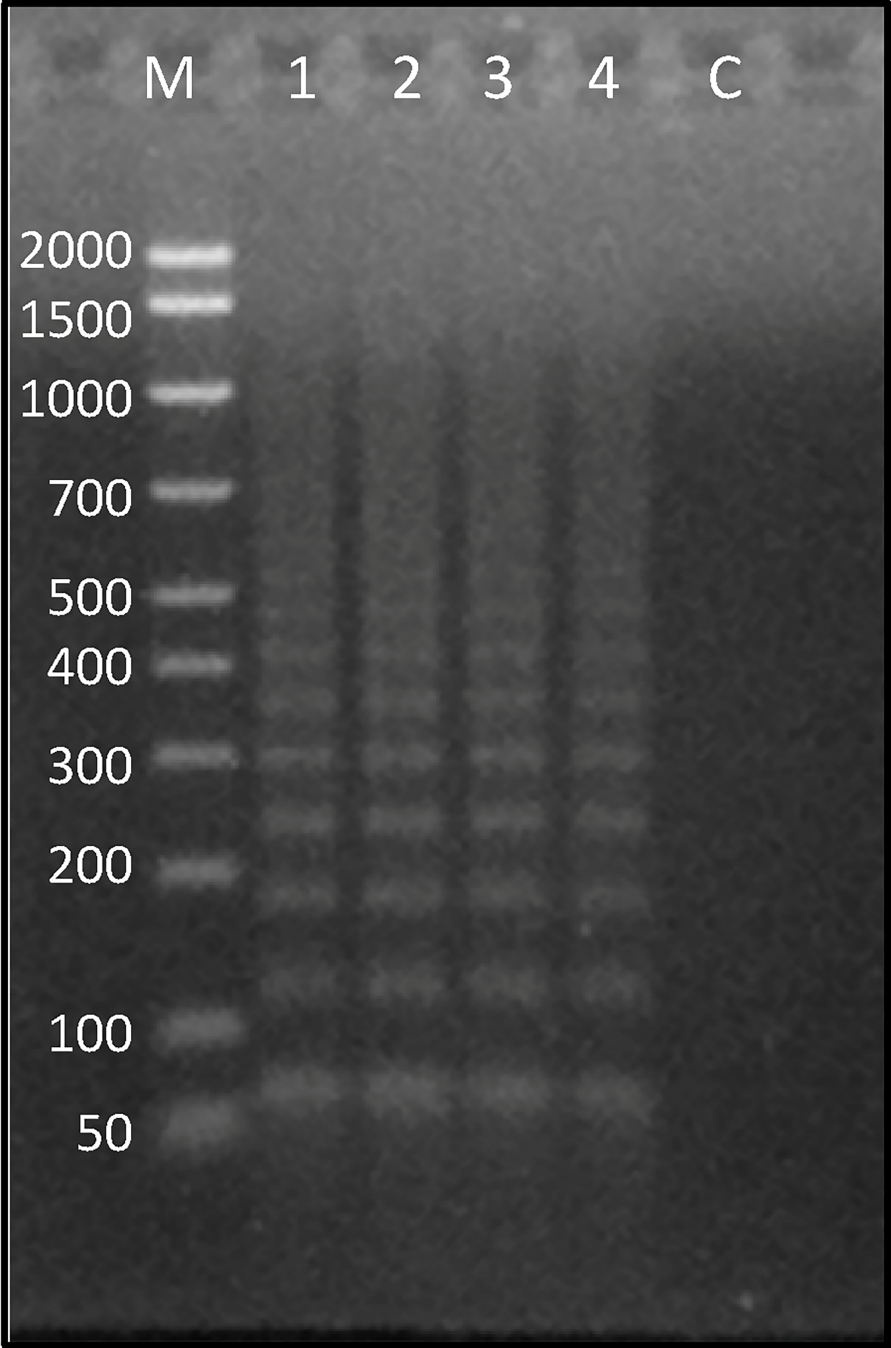
Assessment of Ac4p3 transcription. Agarose gel electrophoresis of Ac4p3 PCR performed on cDNA synthetized from RNA extracted from *A*. *colbecki* and *C*. *islandica* muscle and gonad. M: marker; 1: *A*. *colbecki* muscle; 2: *A*. *colbecki* gonad; 3: *C*. *islandica* muscle; 4: *C*. *islandica* gonad; C: negative control.

The assessment of transcriptional activity of the Ac4p3 element in total RNA extracted from gonad and muscle of *A*. *colbecki* and *C*. *islandica* showed that this element is transcribed in both species (Fig 7).

**Fig 8:**
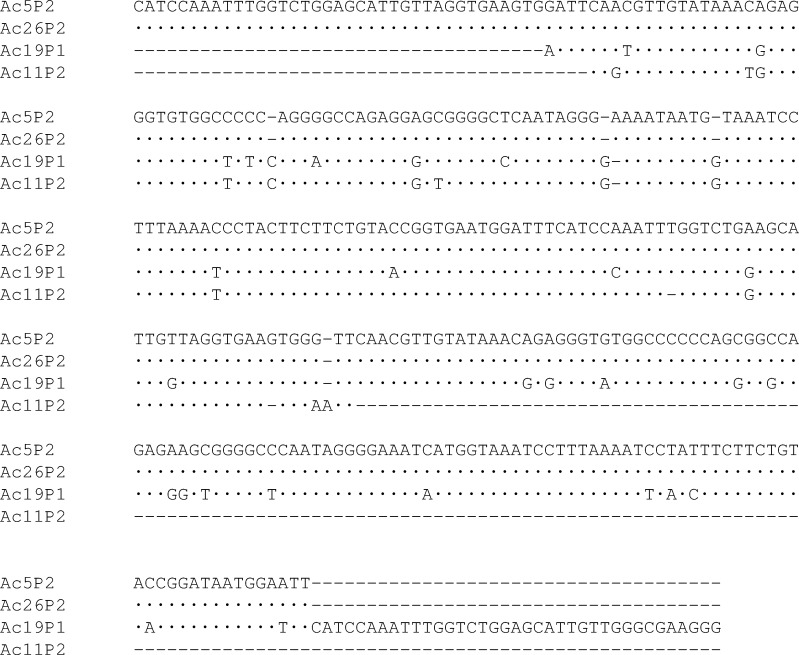
Alignment of partial CvA sequences isolated in *A*. *colbecki*. Alignment of the inserted sequences showing similarity to repeated core of CvA element.

The inserts of four sequenced clones revealed a significant similarity (approximately 70%) to the CvA transposon in other scallops, such as *C*. *farreri*, *Pecten maximus*, *Mizuhopecten yessoensis*, and *Patinopecten magellanicus*. One of these (Ac11p2) was 156 bp long, with an A/T content of 53.84%; two (Ac5p2 and Ac26p2) were 312 bp long, with an A/T content of 54.16%; and one (Ac19p1) was 314 bp long, with an A/T content of 52.86% (Fig 8). The sequence analysis revealed the presence of dimers in these last three insert sequences.

The CvA element described by Gaffney et al. [35] consists of distinct structural modules: the 5’ end is approximately 150 bp in length and is followed by a core region made up of a variable number of copies between 2 and 6 of a 156 bp sequence. The first monomer is truncated at the 5’ end. To confirm that the sequences isolated in *A*. *colbecki* belong to the repetitive core characteristic of the CvA element, a portion of the upstream region was isolated by PCR from cDNA using a forward primer designed based on the alignment of *C*. *farreri* CvA sequences and a reverse primer designed based on the sequences isolated in *A*. *colbecki*. The sequence analysis revealed the first monomer of repeated core, truncated at the 5’ end (S1 Fig).

**Fig 9:**
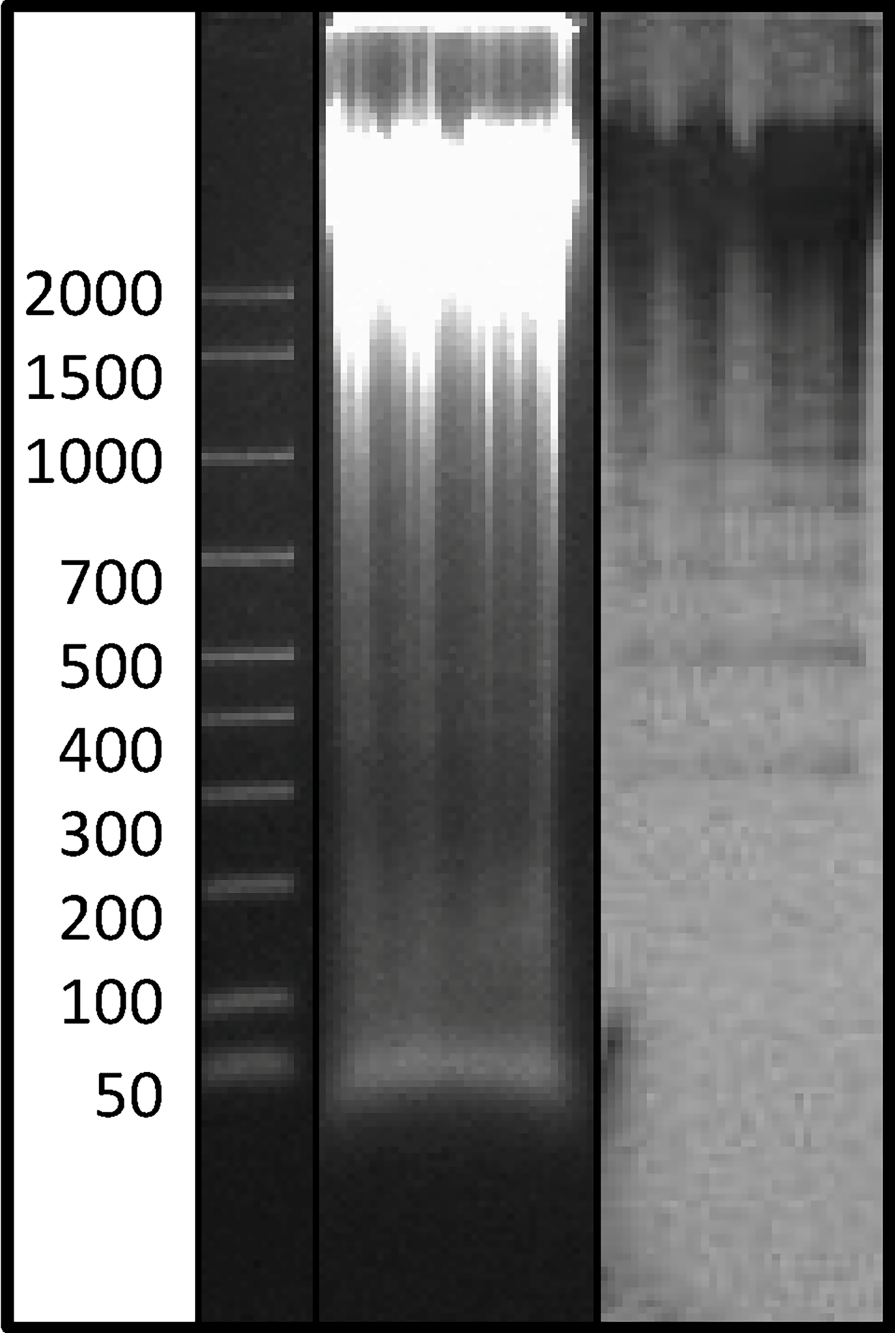
Southern blotting analysis using the Ac19p1 probe. Genomic DNA of *A*. *colbecki* digested with *Eco* RI and Southern hybridization pattern obtained using Ac19p1 as probe.

**Fig 10:**
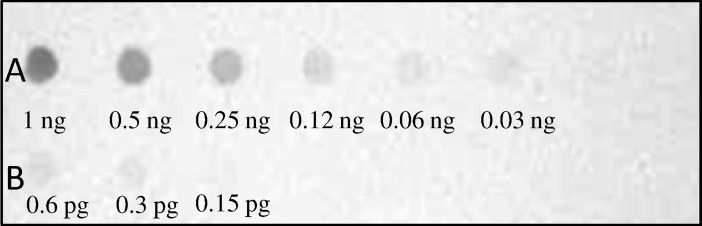
Quantitative dot blot hybridization using the Ac19p1 probe. In row A, increasing amounts of *A*. *colbecki* genomic DNA are spotted from left to right. In row B, increasing amounts of the Ac19p1 sequence are spotted from left to right.

The Southern hybridization pattern obtained using Ac19p1 as a probe showed a characteristic ladder of tandemly repeated elements (Fig 9), and quantitative dot blot analysis indicated that this element accounts for approximately 1% of the genome of *A*. *colbecki* (Fig 10).

**Fig 11:**
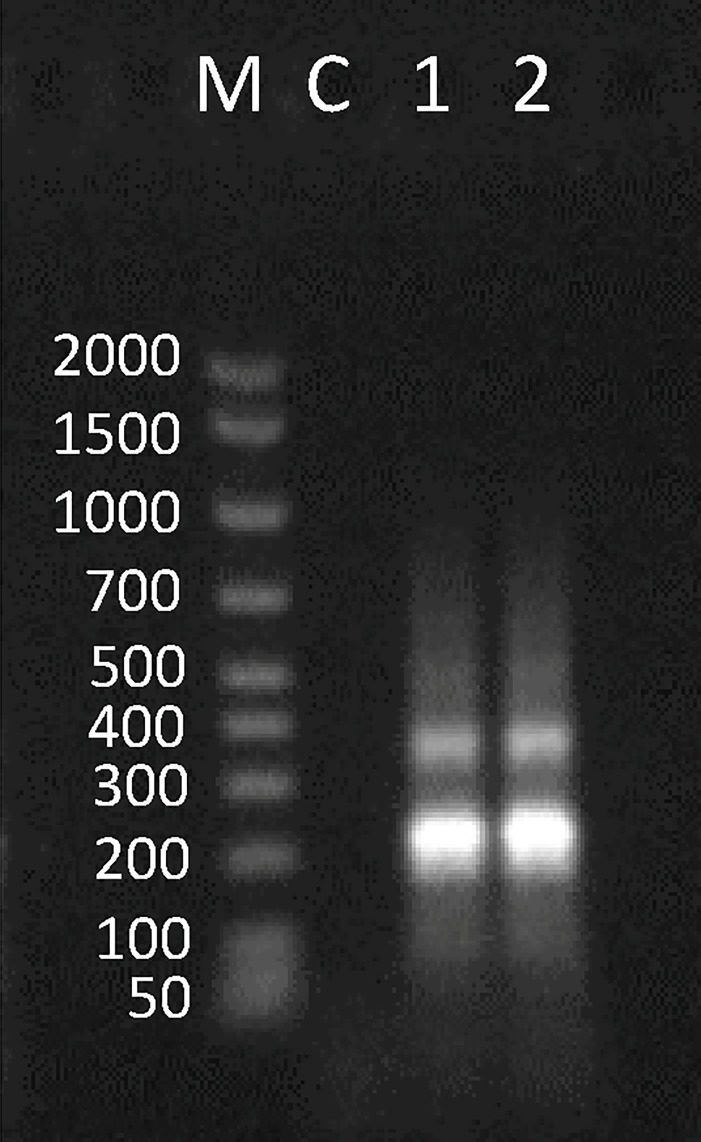
Assessment of CvA transcription. Agarose gel electrophoresis of CvA PCR performed on cDNA synthetized from RNA extracted from muscle and gonads of *A*. *colbecki*. M: marker; C: negative control; 1: *A*. *colbecki* muscle; 2: *A*. *colbecki* gonad.

PCR of cDNA extracted from the gonad and muscle of *A*. *colbecki* indicated transcriptional activity of the CvA element in this bivalve in both analysed tissues (Fig 11).

## Discussion

Repetitive DNA has been characterized in a large number of eukaryotic organisms and represents the main force that influences the size, composition and evolution of genomes [1,3,57]. The study of the structure and organization of repetitive DNA is a useful tool for understanding genomic diversity and the mechanisms leading to the evolution of DNA sequences. In the Antarctic scallop *A*. *colbecki*, we report the presence of two novel repeated elements, Ac4p1 and Ac4p3, which differ in monomer length, structure and abundance in the genome. Southern blotting hybridization revealed a tandem arrangement typical of satellite DNA for Ac4p3, while Ac4p1 exhibited a more complex genomic organization due to the presence of a variable number of subrepeats. The presence of subrepeats has been found in several families of satellite DNA identified in both animal and plant species [58–60]. Monomers consisting of subrepeat units were also found in satDNAs isolated in other molluscan species, such as *Donax trunculus* [30], *A*. *colbecki* [33], *P*. *maximus* [40], and *Nuttallochiton mirandus* [20]. In general, subrepeats affect the evolution of the monomeric sequence through molecular mechanisms such as unequal crossing-over and/or replication slippage that, acting at the subrepeat level, can lead to the formation of new monomeric units called higher order repeat units (HOR units). The spreading of these new variants throughout the genome is due to amplification mechanisms whose efficiency is responsible for the abundance of a given variant. The low abundance of Ac4p1 and Ac4p3 may be related to the low efficiency of the amplification mechanisms acting on these sequences in the *A*. *colbecki* genome.

Another interesting finding from the Southern blotting analysis was the monomer length of ~726 bp for Ac4p1 and ~237 bp for Ac4p3. Satellite DNAs are often characterized by a monomer length of approximately 150–180 bp and 300–360 bp, an important requirement for wrapping the sequence around one or two nucleosomes [61]. This organization has been linked to heterochromatin condensation processes at the centromere [62]. Therefore, the fact that the monomers of the isolated elements here are not of these lengths suggests that they probably do not interact with histone proteins and therefore do not play a role in the chromatin structural organization. This finding may also be in agreement with quantitative data showing a low abundance of both isolated elements in the *A*. *colbecki* genome.

Qualitative dot blot analysis performed in other pectinids and in other polar species revealed the presence of Ac4p3 in species adapted to cold environments. In ectothermal organisms, temperature is one of the principal environmental variables that can drive adaptive evolution and consequently influence the evolution of repetitive DNA. In insects, Feliciello and colleagues [21] suggested a role for DNA methylation of satellite DNA as an epigenetic mechanism responsible for environmental adaptation. The correlation between repetitive DNA and environment was also suggested in our previous work [20], in which we have reported the presence of two satDNA families isolated from the Antarctic chiton *Nuttallochiton mirandus* in polar species. To verify the hypothesis of a correlation between Ac4p3 and temperature, we used specific primers to search for the presence of this element in two pectinids, *C*. *islandica* and *C*. *varia*. The first lives in the Arctic waters in environmental conditions similar to *A*. *colbecki*; the second species lives in a temperate environment and is more closely phylogenetically related to *C*. *islandica* than to *A*. *colbecki* [55,56]. The analysis confirmed the presence of a homologous satDNA only in *C*. *islandica*, supporting the hypothesis of a correlation between repetitive DNA and abiotic factors such as temperature.

Moreover, in the qualitative dot blot, the hybridization signals obtained in bivalves and gastropods using Ac4p3 as a probe suggest that this element was present in the genome of their common ancestor dating its origin over 500 million years.

Recently, several studies have accumulated about the transcriptional activity of satellite DNA, challenging the view of this DNA as “junk" [9,63–65]. In this respect, our analyses showed that Ac4p3 is transcribed in the gonads and muscles of *A*. *colbecki* and *C*. *islandica*. In *Schizosaccharomyces pombe*, *D*. *melanogaster*, *Mus musculus* and *Homo sapiens*, repeated pericentromeric elements generate small interfering RNAs (siRNAs) that interact with the RNAi-induced transcriptional silencing complex (RITS) and affect heterochromatic gene silencing at the centromeric and pericentromeric levels [66]. This mechanism requires low levels of transcription and is considered universal because similar mechanisms have been described in several species [67–69]. The short subrepeat structure, the low abundance of the Ac4p3 element in the *A*. *colbecki* genome and the transcription of this element are three aspects that support the hypothesis of its involvement in a similar mechanism.

The screening of the *A*. *colbecki* genomic library also identified a sequence showing homology to the CvA transposon, which was first discovered in *Crassostrea virginica* [35]. This element was classified as belonging to a new MITE-like family of non-autonomous transposable elements. Its structure shows subterminal inverted repeats, a tandemly repeated core element, and a tetranucleotide microsatellite region. Sequence homologies to the tandemly repeated core have been identified in several bivalve molluscs [14,29,36,40,45]. López-Flores et al. [36], studying this element in oyster genomes, advanced the hypothesis of the existence of an ancient transposable element that acted as the generating element of satellite DNAs in bivalve molluscs. The link between the onset and spreading of satDNAs and transposable elements has also been hypothesized by Šatović and Plohl [45] when discussing the *Pearl*-related sequences identified in *D*. *trunculus*. Overall, an increasing number of studies have linked TEs with satDNAs, even if these two types of repetitive elements differ in their structure, localization, and sequence dynamics. In plants, retroelements from the gypsy superfamily are believed to be responsible for the origin of many centromeric satDNAs [70–72]. In animals, several classes of TEs have been related to satDNAs [35,45,73–75]. The evolutionary relationships between TEs and satDNAs are still unclear, and one hypothesis suggests that TE expansion may represent a source of satDNAs [35,71,76]. On the other hand, the interspersed distribution of TEs might be linked to inverted repeats formed by inversion of satDNA monomers [77]. MITEs are non-autonomous elements lacking an open reading frame (ORF) and can be transcribed if associated with a gene, as commonly observed in plants [78,79]. This association has also been related to a role of MITEs in gene regulation and genome evolution. Intriguingly, our analysis showed that the CvA element in *A*. *colbecki* is transcribed in both analysed tissues. This finding, together with the wide distribution of CvA in bivalves, suggests a possible role of this element in shaping the genomes of these molluscan species.

## Conclusions

The results obtained here allowed us to expand our knowledge of the bivalve repetitive DNA fraction. In particular, our analyses in *A*. *colbecki* evidenced that this satDNA is widespread among polar molluscs, indicating a possible link between repetitive DNA and abiotic factors. Moreover, the presence of this element in bivalves and gastropods suggests that this element was present in the genome of their common ancestor, dating its origin at over 500 mya. The transcriptional activity of Ac4p3 satDNA and the CvA transposon indicates a possible role of these ancient elements in shaping molluscan genome architecture.

## Supporting information

S1 Fig Partial sequence of the CvA element in *A*. *colbecki*. The sequence contains a portion of the region (in bold) upstream of the repeated core, followed by the first repeat element, truncated at the 5’ end.
(JPG)
