# Supplementary figures and images for: New insights into the genome repetitive fraction of the Antarctic bivalve Adamussium colbecki
Source: PLoS One. 2018 Mar 28;13(3):e0194502. doi: 10.1371/journal.pone.0194502 (PMC5874043; doi:10.1371/journal.pone.0194502)

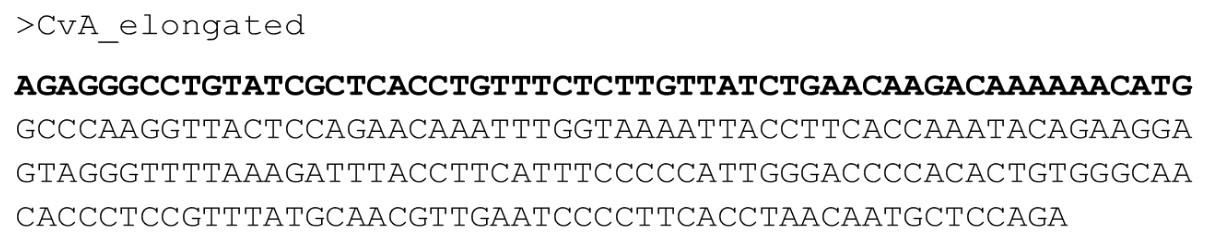

Supplement: S1 Fig — The sequence contains a portion of the region (in bold) upstream of the repeated core, followed by the first repeat element, truncated at the 5’ end. (JPG) [file pone.0194502.s001.jpg]
